# Maxillofacial Injuries Sustained by Riders of Electric-Powered Bikes and Electric-Powered Scooters

**DOI:** 10.3390/ijerph192215183

**Published:** 2022-11-17

**Authors:** Shimrit Arbel, Elad Zrifin, Reema Mahmoud, Eitan Mijiritsky, Leonid Groysman, Amir Shuster, Heled Rahima, Shlomi Kleinman, Clariel Ianculovici, Oren Peleg

**Affiliations:** 1 Department of Otolaryngology Head and Neck Surgery and Maxillofacial Surgery, Tel-Aviv Sourasky Medical Center, Tel Aviv 6423906, Israel; 2 Maurice and Gabriela Goldschleger School of Dental Medicine, Tel Aviv University, Tel Aviv 6423906, Israel

**Keywords:** electric bikes, electric scooters, maxillofacial trauma, electric powered vehicles, maxillofacial injuries

## Abstract

Objectives: The purpose of our study is to retrospectively analyze and compare the patterns of maxillofacial-related injuries among rides of electric-powered bikes (E-bikes) and electric-powered scooters (E-scooters), the associated risk factors, and the required treatment. Materials and methods: The medical files of all riders presenting to the emergency department at the Tel Aviv Sourasky Medical Center between 2019 and 2020 with oral- and maxillofacial-related injuries due to E-bike and E-scooter accidents were reviewed. Results: A total of 320 riders sustained oral- and maxillofacial-related injuries due to trauma involving E-bikes and E-scooters during the study period. E-scooter riders were involved in 238 accidents (74.5%) while E-bike riders accounted for the remaining 82 accidents (27.5%). Eighty-four out of 320 riders (26.25%) were hospitalized and required surgical interventions. Most of the 232 riders (72.5%) who reported not wearing a protective helmet during the index accident were E-scooter riders. In addition, 39 riders (18.66%) were riding either of these electric-powered vehicles under the influence of alcohol. Conclusions: E-bike riders are more likely to sustain a maxillofacial fracture than E-scooter riders. Not wearing a protective helmet and riding under the influence of alcohol are major risk factors for maxillofacial injuries.

## 1. Introduction

Recent years have witnessed the increasing popularity of electric-powered bicycles (E-bikes) and electric-powered scooters (E-scooters) as a mode of transportation worldwide. Rental E-scooters were first introduced in Israel as an easily accessible and inexpensive means of urban transportation in early 2019. The current estimated number of E-scooters in Israel is between 100,000 and 150,000, of which around 2000 rental E-scooters are distributed via four major providers throughout the city of Tel Aviv alone. Additionally, thousands of private E-bikes and E-scooters are being used throughout the country [1,2]. Riders may have insufficient experience and no proper training in handling these relatively new means of transportation, along with insufficient use of protective gear, especially those using rental E-scooters. Furthermore, a designated infrastructure for these means of transportation is not widespread throughout urban areas, forcing the users to navigate either between motor vehicles or among pedestrians. Concern regarding potential injury to riders and pedestrians has risen markedly.

An analysis by the US national electronic injury surveillance system from 2000 to 2017 revealed differences in the patterns of use and injury among riders of E-bikes and E-scooters compared to those of traditional pedal-operated bicycle riders [3]. In China, the E-bike-related nonfatal injury rate increased almost four-fold and the mortality rate increased six-fold from 2004 to 2010 [4]. In Israel, there was a six-fold increase in the number of hospitalized patients due to E-bike and E-scooter incidents from 2013 to 2015 [5]. Goh et al. recently observed that accidents involving E-bikes and E-scooters are contributing to health, economic, and social problems worldwide [6].

A study of vehicular trauma that was conducted in Israel between 2014–2019 found that accidents involving E-bikes and E-scooters were responsible for more than 10% of the hospital admissions for dental and maxillofacial injuries [7]. We considered that a data analysis of the extent of these injuries may quantify their influence on emergency department (ED) admissions and burden on healthcare systems. The aim of the current retrospective study, therefore, was to analyze the patterns of maxillofacial-related injuries caused by E-bike and E-scooter use in the Tel Aviv area and the associated treatments of riders admitted to the ED of the Tel Aviv Sourasky Medical Center (TASMC), Israel.

## 2. Materials and Methods

### 2.1. Study Design and Participants

After obtaining the approval of the institutional ethics committee (Helsinki 0552-21-TLV), the medical files of all riders who presented to the TASMC ED with oral- and maxillofacial-related injuries due to E-bike and E-scooter accidents between 2019 and 2020 were screened. Riders admitted to the ED after sustaining an injury while riding non-electric bikes or scooters and pedestrians who sustained an injury caused by E-bikes or E-scooters were excluded from the study.

### 2.2. Data Collection

Data extracted from each rider’s medical record included demographic characteristics: age, gender, comorbidities, parameters related to the accident (type of vehicle (E-bike or E-scooter)), alcohol blood levels, use of protective gear, parameters related to the medical treatment (whether a computed tomographic (CT) scan was performed), type and location of dental and/or maxillofacial injury, additional concomitant injuries, type of surgical intervention, and the need for hospitalization.

In addition to soft-tissue lacerations and abrasions, injuries involving the facial bones were further subclassified according to the Craniomaxillofacial AO Surgery reference [8]:

- Minor injuries (dentoalveolar injuries and facial lacerations);
- Mandibular fractures (symphysis/parasymphysis, angle/body, condylar/subcondylar);
- Midface fractures (orbit, naso-orbito-ethmoidal [NOE], zygomatic complex/arch, LeFort I/II/III, maxilla);
- Skull base and cranial vault fractures (frontal bone and sinus).
- The surgical interventions were subdivided into:
- Suturing;
- Splinting;
- Closed reduction approaches (either intermaxillary fixation or Gillies approach);
- Open reduction and internal fixation (ORIF).

### 2.3. Statistical Analysis

Categorical variables were summarized as frequency and percentage. Age was evaluated for normal distribution by a histogram and the Kolmogorov–Smirnov test. Since age was not normally distributed, it was reported as median and interquartile range (IQR). Categorical variables were compared with the Chi-squared test and Fisher’s exact test, and age was compared by the Mann–Whitney test. Logistic regression analyses were used to estimate the relationship between risk factors (such as the use of a protective headgear and driving under the influence of alcohol), type of treatment performed, age of group, type of injuries, and type of ride. All statistical tests were two-sided. A *p*-value < 0.05 was considered statistically significant. SPSS 2020 software was used for all statistical analyses (“IBM SPSS Statistics for Windows, Version 27.0, IBM Corp., Armnok, NY, USA, 2020”).

## 3. Results

### 3.1. Rider Characteristics

Rider details and characteristics are presented in Table 1. A total of 320 riders with oral- and maxillofacial-related injuries due to trauma involving E-bikes and E-scooters were admitted to the TASMC ED between 2019 and 2020. They included 193 males (60.3%) and 127 females (39.7%), with a median age of 28.84 years. E-scooter riders were involved in 238 accidents (74.4%) and E-bike riders accounted for the remaining 82 accidents (25.6%). The E-bike group was older than the E-scooter group (median age 32.28 years vs. 27.66 years, respectively, *p* = 0.008). Additionally, age was 1.1-fold associated with frontal (95% CI 1–1.22, *p* = 0.04) and NOE fractures (95% CI 1.01–1.21, *p* = 0.03).

Eighty-four of the 320 riders (26.25%) were hospitalized and required surgical interventions. The remaining 236 riders (73.75%) were diagnosed as having minor injuries (i.e., soft-tissue and dentoalveolar) or injuries that did not require further intervention, and were therefore discharged after receiving appropriate treatment in the ED by an oral and maxillofacial surgeon. Most of the 158 riders (49.4%) who reported not wearing a protective helmet during the index accident were E-scooter riders. The blood alcohol levels of 209 riders (65.3%) were examined, and 39 of them (18.66%) were found to have driven these electric-powered vehicles under the influence of alcohol.

### 3.2. Type of Injuries and Treatment

**Figure 1:**
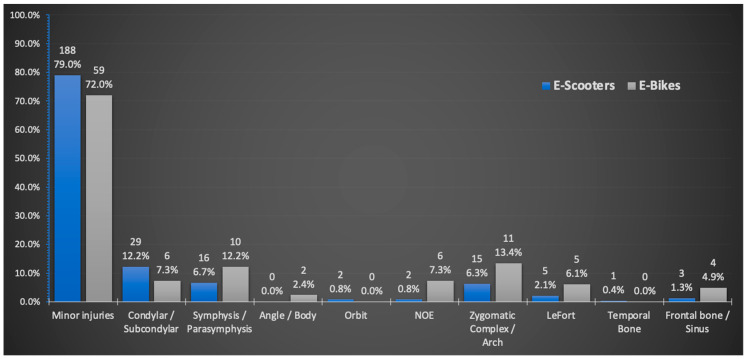
Distribution of injuries among E-scooters’ and E-bikes’ riders.

The distribution of type of injuries and their locations are shown in Figure 1. In total, 188 E-scooter riders (79%), and 59 E-bike riders (72%) sustained minor injuries, such as dentoalveolar wounds and facial lacerations, and 30% of the riders in each group required suturing and/or splinting. Mandibular fractures accounted for most of the facial bone fractures in both groups, while condylar and subcondylar fractures were more frequent in the E-scooter group. The numbers of zygomatic complex fractures and NOE fractures were significantly higher in the E-bike group compared to the E-scooter group (*p* = 0.042 and *p* = 0.004, respectively). E-bike riders were 6.03 times more likely to be diagnosed with zygomatic fracture than E-scooter riders (95% CI 1.52–23.92, *p* = 0.01). Skull base and cranial vault fractures were seen only in the E-scooter group and among the riders of both groups who did not wear protective headgear. More riders in the E-bike group required hospitalization after injury (*p* = 0.03), although the need for ORIF was significantly higher among E-scooter riders (*p* = 0.039).

### 3.3. Protective Headgear Use

The use of protective headgear among the E-bike and E-scooter riders is presented in Table 2. Headgear use was recorded in 246 of the 320 enrolled riders (76.8%). Eighty-eight riders reported wearing a protective helmet during the index accident (35.7%), compared to 158 that reported not doing so (64.23%). There was no significant difference in the rider’s age regarding protective headgear use. In addition, there was no significant difference in injury location, type of treatment, need for hospitalization between riders who did and did not wear protective headgear. Alcohol blood levels were significantly higher among the latter riders (*p* = 0.01).

### 3.4. Driving under the Influence

Blood alcohol levels were recorded for 209 (65%) riders (Table 3). The levels of 39 of them (18.66%) were high, and most were E-scooter riders (79.5%). Only 6 (16.7%) intoxicated riders were wearing protective headgear compared to 51 (39.8) of the sober drivers (*p* = 0.01). Moreover, inebriated riders not only sustained more frontal sinus and zygomatic bone fractures than non-inebriated ones (*p* = 0.046 and *p* = 0.044, respectively), but significantly more of them required hospitalization (*p* = 0.004) and more extensive surgical intervention compared to riders with normal blood alcohol levels (*p* = 0.047 and *p* = 0.016). Driving under the influence of alcohol was associated with 19.97- and 4.9-times increased risk of frontal and zygomatic bone fractures, respectively (95% CI 1.1–361.02, *p* = 0.04 and 95% CI 1.21–19.79, *p* = 0.03), 2.73 times increased need for hospitalization (95% CI 1.21–6.16, *p* = 0.02) and 3.7 times increased risk of undergoing ORIF procedures (95% CI 1.24–11.03, *p* = 0.02).

## 4. Discussion

E-bikes and E-scooters represent a new means of urban transportation, especially in large metropolises. Their gradual rise in popularity has been attributed to their convenience, affordability, and status as a “green” alternative to vehicles with combustion engines [9]. There are, however, many risk factors associated with their use. Such risk factors include ride-related variables, such as age and riding experience, infrastructure factors, such as uneven pavements and lack of designated lanes, vehicular factors, such as low visibility (by being small and quiet vehicles), being driven at a relatively high speed, as well as a lack of structural stability due to being lightweight, having small wheels, and having a low level or no suspension in comparison to larger two-wheel vehicles, such as motorcycles. This gradual rise in use, taken together with the high number of risk factors associated with riding E-bikes and E-scooters, has led to an increase in craniomaxillofacial-related injuries, leading to a substantial burden on the healthcare system, especially in the EDs.

A U.S. national database showed that facial and head injuries from micro-mobility devices have tripled in the last 10 years [9]. Additionally, studies have shown that E-bike- and E-scooter-related crashes have a higher rate of facial soft-tissue injuries as well as a higher rate of dental injuries and facial fractures when compared to non-electric powered bicycle crashes [10]. In Israel, there was a 13-fold increase in the number of hospitalized patients due to E-bike and E-scooter accidents from 2013 to 2019 [7]. The information on E-bike- and E-scooter-related injuries in Israel, however, is scarce.

Our current study sample included 320 riders admitted to the ED during 2019–2020 after sustaining craniomaxillofacial-related injuries due to E-bike and E-scooter accidents. E-scooter riders represented a higher percentage of the patients in our study (238 riders, 74.4%). This might be related to the vast distribution of rental E-scooters, which leads to a higher percentage of new or infrequent users, as opposed to E-bikes which are used more often. A study on injuries related to electric scooter and bicycle use in Washington DC found that injured cyclists tend to be frequent users of bikes for utilitarian reasons, while injured E-scooter riders are more often infrequent users who are riding for social reasons [11]. Those authors also observed that E-scooter riders were significantly younger than E-bike riders. DiMaggio et al. also observed that persons injured when using E-bikes were considerably older than those who used E-scooters [3].

E-scooters and E-bikes differ greatly in their structure, stability, and speed. Unlike E-bikes, the users of E-scooters stand on the vehicle while riding and will fall free from the vehicle, thereby absorbing the entire impact of the fall when they lose their balance in case of accident. This may affect the mechanism of injury and its severity. Instability or a falling sensation triggers the neuromuscular protective reflexes, causing the arms to extend in an attempt to break the fall, in order to reduce the severity of injury and the impact force. This mechanism may protect the head, thorax, abdomen, and pelvis [12]. One study on mountain-biking-related injuries treated in EDs in the United States found that greater proximal upper extremity injuries to cyclists could reflect injuries sustained when thrown from bicycles [13]. On the other hand, lower distal extremity injuries are seen more often in E-scooter riders, which could result from the riders’ attempt to stop the fall using their feet [11].

We assume that the lack of ability to brace for a fall using the extremities may lead to craniomaxillofacial injuries. Our assumption is supported by the findings of a study by Shiffer et al., who demonstrated that the odds of having a distal lower extremity injury in E-scooter riders was significantly decreased among those with a craniomaxillofacial injury [12]. The etiology of craniomaxillofacial trauma is diverse, with road traffic accidents, assaults, and falls being the leading causes cited in the maxillofacial literature [14]. The type and location of the injury are directly affected by the surrounding conditions. In general, mandibular fractures, followed by zygomatic complex and nose fractures, are reportedly the most common types of facial fractures [14]. This correlates with our study findings, in which minor injuries were the most common type of injury (67.8%), followed by mandibular fractures (17.31%), midface fractures (12.64%), and cranial vault fractures (2.2%). Midface fractures were significantly higher in the E-bike group than in the E-scooter group. In addition, a significantly higher percentage of E-bike riders required hospitalization for ORIF compared to E-scooter riders, which may reflect the severity of their injuries. These results correlate with those of other studies that compared both vehicles and found that injuries involving E-bikes were more severe than injuries involving E-scooters [11,15]. Tefft et al. found that as a motor vehicle increases in speed, the risk of serious injury or fatality for vulnerable drivers also increases because of their reduced field of vision [16]. In a study on the usage patterns of E-bikes and E-scooters, Almannaa et al. demonstrated that the average speed of E-bikes (3:01–3:44 m/s) was higher than the average speed of E-scooters (2:19–2:78 m/s), leading those authors to suggest that higher speed may be a primary contributing factor to the severity of injury [17].

These results are further supported by comparisons made between powered and non-powered bikes. Van der zaag et al. [18] compared maxillofacial injuries among 238 regular bicycle and 73 E-bike riders. They found that although alcohol use was higher in the conventional bicycle group (32% vs. 16%), E-bikers sustained midfacial fractures more frequently (47% vs. 34%). Gülses et al. [19] collected data from 162 injured bicycle riders, 86 of them presented with at least one maxillofacial fracture. The overall ratio of the number of the fracture line/fracture case was 2.80. However, this ratio was statistically higher among E-bike riders (4.25) compared to non-E-bike riders (2.34).

Interestingly, compared to non-powered bike cyclists, E-scooter and E-bike riders are less likely to use a protective helmet [10]. Although data regarding patterns of using a protective headgear were recorded in 246 out of the 320 riders who presented to our ED, only 88 of them (35.7%) stated that they did so during the index accident. There was no significant difference in protective helmet use between E-bike and E-scooter riders. The fact that there were no differences in injury location, type of treatment, or need for hospitalization between riders who did and did not wear protective headgear may be related to the commonly used half-shell helmet type, which may protect only the cranial vault.

The blood alcohol level was recorded in 209 out of the 320 riders who presented to our ED with oral and maxillofacial related injuries due to E-bike and E-scooter accidents. Alcohol intoxication may cause depression of the neuromuscular protective reflexes that help brace the fall during an accident by extending the extremities. Direct trauma to the head is more likely to occur due to diminished reflexes and result in facial bone fractures. Studies have shown that alcohol negatively influences visual and auditory functions, reaction time, vigilance, tracking, and attention [12]. We found that drivers with too-high blood alcohol levels were less likely to wear protective headgear, were more likely to be admitted to hospital care due to serious injuries and required significantly more corrective surgeries in comparison to non-intoxicated drivers. These finding are compatible with those of others who reported that patients with craniomaxillofacial injuries were 10 times more likely to have been intoxicated [12].

## 5. Conclusions

The increasing use of E-bikes and E-scooters without proper rider-training and proper observance of precautions, and the lack of a designated infrastructure, is associated with an alarming rise in serious maxillofacial injuries. E-bike riders are more likely to sustain a maxillofacial fracture than E-scooter riders. Not wearing protective headgear or wearing only a half-shell helmet while riding, as well as riding under the influence of alcohol, are major risk factors of accidents leading to maxillofacial injuries. Based upon our study results, we recommend the use of a full-face protective helmet while riding electric-powered vehicles and prohibiting the use of these vehicles while under the influence of alcohol. Education, training, and designated traffic lanes may go far in reducing the number and extent of these injuries.

## Figures and Tables

**Table 1:** Differences between E-bikes and E-scooters riders (n = 320 patients).

	E-Scooters	E-Bikes	*p*-Value
Patients (%)	238 (74.4%)	82 (25.6%)	-
Age (years) ^#^	27.66 (8)	32.28 (18)	0.008
X-ray (%)	153 (64.3%)	58 (70.7%)	0.288
Protractive helmet (%)	62 (34.1%)	26 (40.6%)	0.346
Blood alcohol levels above normal (%)	31 (20.4%)	8 (14%)	0.293
Additional injuries (%)			
TBI	14 (5.9%)	4 (4.9%)	>0.999
Orthopedic	112 (47.1%)	44 (53.7%)	0.302
Chest	3 (1.3%)	1 (1.2%)	>0.999
Combinations	3 (1.3%)	1 (1.2%)	>0.999
Need for hospitalization (%)	55 (23.1%)	29 (35.4%)	0.03
Surgical intervention (%)			
Suturing or Wire splinting	71 (29.8%)	22 (26.8%)	0.606
Closed treatment (IMF/Gillies)	26 (10.9%)	8 (9,8%)	0.767
ORIF	21 (8.8%)	14 (17.1%)	0.039
Combinations	11 (4.6%)	5 (6.1%)	0.567
Follow-up (%)	81 (34%)	35 (42.7%)	0.16

# Expressed as median (IQR). Abbreviations: TBI, traumatic brain injury; IMF, intermaxillary fixation; ORIF, open reduction and internal fixation.

**Table 2:** Differences between headgear-wearers and non-headgear-wearers (n = 246 patients).

	Headgear Wearers	Non-Headgear- Wearers	*p*-Value
Patients (%)	88 (35.77%)	158 (64.23%)	-
Age (years) ^#^	29.05 (11.75)	27.78 (10)	0.485
X-ray (%)	58 (65.9%)	109 (69%)	0.62
Blood alcohol levels above normal (%)	6 (10.5%)	30 (28%)	0.01
Additional injuries (%)	49 (55.7%)	74 (46.8%)	0.231
Need for hospitalization (%)	22 (25%)	47 (29.7%)	0.427
Intervention (%)			
Suturing or Wire splinting	27 (30.7%)	47 (29.7%)	0.878
Closed treatment (IMF/Gillies)	12 (13.6%)	20 (12.7%)	0.827
ORIF	8 (9.1%)	22 (13.9%)	0.267
Combinations	6 (6.8%)	9 (5.7%)	0.724

# Expressed as median (IQR). Abbreviations: TBI, Traumatic brain injury; IMF, Intermaxillary fixation; ORIF, Open reduction and internal fixation.

**Table 3:** Differences between driving under the influence and sober drivers (n = 209 patients).

	Drunk Drivers	Sober Drivers	*p*-Value
Patients (%)	39 (18.67%)	170 (81.33%)	-
Age (years) ^#^	29.87 (10)	29.3 (13)	0.635
Injury Location (%)			
Minor injuries *	21 (53.8%)	134 (78.8%)	0.002
Condylar/subcondylar	4 (10.3%)	20 (11.8%)	0.79
Symphysis/parasymphysis	4 (10.3%)	13 (7.6%)	0.591
Angle/body	0 (0%)	1 (0.6%)	0.631
Orbit	1 (2.6%)	1 (0.06%)	0.253
Frontal sinus	3 (7.7%)	3 (1.8%)	0.046
Zygomatic complex	6 (15.4%)	10 (5.9%)	0.044
LeFort	1 (2.6%)	5 (2.9%)	0.899
NOE	3 (7.7%)	4 (2.4%)	0.095
Protractive helmet (%)	6 (16.7%)	51 (39.8%)	0.01
Need for hospitalization (%)	18 (46.2%)	40 (23.5%)	0.004
Intervention (%)			
Suturing or Wire splinting	8 (20.5%)	49 (28.8%)	0.327
Closed treatement (IMF/Gillies)	8 (20.5%)	15 (8.8%)	0.047
ORIF	8 (20.5%)	12 (7.1%)	0.016
Combinations	4 (10.3%)	6 (3.5%)	0.093

^#^ Expressed as median (IQR). * Lacerations/Dentoalveolar. Abbreviations: IMF, Intermaxillary fixation; ORIF, Open reduction and internal fixation.

## Data Availability

The data that support the findings of this study are available on r quest from the corresponding author. The data are not publicly available due to ethical and legal restrictions.
